# The association of liver function and quality of life of patients with liver cancer

**DOI:** 10.1186/s12876-019-0984-2

**Published:** 2019-05-02

**Authors:** Leung Li, Frankie Mo, Edwin P. Hui, Stephen L. Chan, Jane Koh, Nelson L. S. Tang, Simon C. H. Yu, Winnie Yeo

**Affiliations:** 1 Department of Clinical Oncology, Prince of Wales Hospital, Faculty of Medicine, The Chinese University of Hong Kong, Shatin, NT Hong Kong SAR; 2 State Key Laboratory in Oncology in South China, Prince of Wales Hospital, Faculty of Medicine, The Chinese University of Hong Kong, Shatin, Hong Kong SAR; 3 Department of Chemical Pathology, Li Ka Shing Institute of Health Sciences, Faculty of Medicine, The Chinese University of Hong Kong, Shatin, Hong Kong SAR; 4 Department of Diagnostic and Interventional Radiology, Prince of Wales Hospital, Shatin, Faculty of Medicine, The Chinese University of Hong Kong, Shatin, Hong Kong SAR

**Keywords:** Hepatocellular carcinoma, Health related quality of life, EORTC QLQ-C30, QLQ-HCC18, C30 index-score, HCC18 index-score, Hepatic function, Correlation, Child-Pugh, MELD, Albumin to alkaline phosphatase ratio, Alkaline phosphatase to platelet ratio, ALBI

## Abstract

**Background:**

Quality of life (QOL) assessments with the European Organization for Research and Treatment of Cancer (EORTC) QLQ-C30, QLQ-HCC18, C30 and HCC18 index scores have been shown to be prognostic factors for overall survival (OS) in patients with hepatocellular carcinoma (HCC), independent of disease stage and liver function. Liver function parameters (including bilirubin, albumin, international normalized ratio [INR], Child-Pugh class, ALBI grade, MELD, alkaline phosphatase [ALP]-to-platelet ratio, albumin-to-ALP ratio) have also been found to be independent prognostic factors for OS in HCC patients. There has been scanty data on whether QOL and baseline liver function per se are correlated in HCC patients. This study investigates the correlations between baseline QOL data and liver function variables in HCC patients.

**Methods:**

From 2007 to 2011, 517 patients were enrolled. Baseline QOL was assessed at diagnosis using the EORTC QLQ-C30 and QLQ-HCC18; thereafter C30 and HCC18 index scores were derived. Clinical and laboratory data were collected. For liver function assessment, Child-Pugh class, ALBI grade, MELD, ALP-to-platelet ratio and albumin-to-ALP ratio were derived. Correlation analyses were performed between QOL and liver function data.

**Results:**

Complete QOL data were available in 472 HCC patients. After adjusting for clinical variables, significant correlations were found between QOL (QLQ-C30 and QLQ-HCC18) and dichotomized liver function variables (including Child-Pugh class, ALBI grade and the presence of ascites). It was demonstrated that QOL had significant and potentially clinically important correlations with continuous liver function variables (albumin, bilirubin, ALP and albumin-to-ALP ratio), with the highest Spearman’s rank correlation coefficient (rho) exceeding 0.4. HCC18 and C30 index scores were also significantly correlated with these liver function variables. HCC18 index score, which had rho up to 0.37, generally performed better than C30 index score, which had rho up to 0.33.

**Conclusions:**

In HCC patients, baseline QOL assessment (using EORTC QLQ-C30, QLQ-HCC18, C30 index-score or HCC18 index-score) is significantly correlated with liver function. Based on the findings of this study, future trials are warranted to assess whether treatment to enhance liver function could improve HCC patients’ QOL.

## Background

Liver cancer ranked the fifth most common cancer in the world, with 782,451 new cases per year in 2012. It was also the second leading cause of cancer death worldwide, responsible for 745,533 deaths in 2012. The ratio of mortality to incidence was 0.95, signifying its very aggressive nature and poor prognosis [1]. Screening population at risk for HCC by regular ultrasonography and alpha fetoprotein (AFP) level has been proven in one study to improve rate of early detection and increase the chances of curative treatment and survival [2]. However, for most parts of the world, only opportunistic screening is offered to patients with chronic liver diseases [3]. Various management guidelines for HCC are available, these include the Barcelona Clinic Liver Cancer staging classification and treatment approach for HCC (BCLC) [4, 5], Asia-Pacific Association for the Study of the Liver guidelines on the management of HCC (APASL) [6], European Association For The Study Of The Liver-European Organization For Research And Treatment Of Cancer clinical practice guidelines on management of HCC (EASL) [7], and the American Association for the Study of Liver Diseases guidelines for the treatment of HCC (AASLD) [8]. These guidelines unanimously recommend that preserved liver function is an important pre-requisite to deliver effective treatment to HCC patients.

Most hepatocellular carcinomas (HCC) arise in patients with cirrhosis. The most frequent etiologies of HCC in western countries have been chronic hepatitis C virus (HCV) infection and alcoholic liver disease [9–13], while in Asian countries, the main etiological factor has been chronic hepatitis B virus (HBV) infection [9, 10, 14]. Regardless of etiology, patients with HCC are frequently challenged by impairment of liver function as a result of chronic liver disease and cirrhosis in addition to liver tumor burden. Inadequate liver functional reserve creates difficulty in clinical management and negatively affect prognosis.

Liver function has been demonstrated to be associated with overall survival (OS) in patients with HCC. Specifically, parameters that have been reported to be independent prognostic factors in HCC include bilirubin [15, 16], albumin [16], international normalized ratio (INR) [17], alkaline phosphatase (ALP) [18], Child-Pugh classification [19, 20], albumin-bilirubin (ALBI) grade [21, 22], Model for End-stage Liver Disease (MELD) [23], ALP-to-platelet ratio [24] and albumin-to-ALP ratio [17, 18, 25].

QOL in HCC patients is a complex issue affected by medical, psychological, social and spiritual factors [26]. While symptoms arising from HCC tumor per se, the underlying chronic liver disease and cirrhosis, as well as therapeutic intervention for HCC might affect patients’ QOL, other aspects including socio-spiritual support, patient’s coping skill, cultural background and health literacy could also influence QOL. Various instruments have been developed to measure QOL in HCC patients.

The European Organization for Research and Treatment of Cancer (EORTC) QLQ-C30 and Spitzer Quality of Life Index have been found to be prognostic of OS in patients with advanced stage HCC [27–29]. Subsequently, the EORTC QLQ-HCC18, a QOL instrument specifically designed to address issues faced by HCC patients, has also been found to be prognostic for OS in unselected HCC patients, independent of HCC stage and liver function [30]. More recently, in an attempt to simplify the interpretation of results obtained from the various functional domains and symptoms/items scores in the EORTC QLQ-C30 and QLQ-HCC18 assessment, we reported two scoring indices, namely the C30 index-score and HCC18 index-score. These are two single summative scores representing QLQ-C30 and QLQ-HCC18 respectively and they facilitate survival analysis. These have been demonstrated to be highly significant factors for OS [30]. On the other hand, Functional Assessment of Cancer Therapy - General (FACT-G) has been evaluated but has not been reported to be prognostic of OS in HCC patients [31].

Although functional impairment of the hepatic organ commonly occurs in HCC patients, there is very limited data on whether baseline liver function and QOL are correlated in HCC patients. We hypothesized that QOL in HCC patients is correlated with the status of their liver function. We, therefore, attempt to assess the correlation between liver function and QOL in HCC patients.

## Methods

### Patients and methods

The study was approved by the Joint Chinese University of Hong Kong - New Territories East Cluster Clinical Research Ethics Committee. From January 2007 to December 2011, all patients with newly diagnosed HCC who attended the Joint Hepatoma Clinic of the Prince of Wales Hospital were invited to this study. Informed written consents were obtained from all participants.

The eligibility criteria included age 18 years or above; newly diagnosed HCC established either by histology, or the combination of radiological and biochemical findings (hypervascular hepatic lesion and elevated AFP ≥400μg/L, or typical pattern in 2 radiological modalities by ultrasonography, multiphasic computed tomography, magnetic resonance imaging or angiography); treatment-naïve for HCC, ability to read and comprehend Chinese. The exclusion criteria included history of other malignancies, encephalopathy or cognitive impairment.

### QOL assessment

The Chinese version of EORTC QLQ-C30 [32] and QLQ-HCC18 [33] questionnaires were used to assess patients’ baseline QOL on the same day upon entering the study. C30 and HCC18 index-scores were calculated as previously published [30]. Detailed descriptions of these are listed in Table 1.

**Table 1:** Quality of life instruments used in the study

QOL instrument	Description
European Organization for Research and Treatment of Cancer QLQ-C30 (EORTC QLQ-C30) [32]	EORTC QLQ-C30 is a general cancer instrument containing multiple items, measured in multiple-point Likert scales, that reflect the multidimensionality of QOL construct [32]. It includes five functional domains (physical, role, cognitive, emotional and social), three symptom domains (fatigue, pain, nausea and vomiting), and a global health and QOL domain. Six single items assess common symptoms in cancer patients (dyspnea, appetite loss, sleep disturbance, constipation and diarrhea) and financial problem. All scales and domains are transformed to scores ranging from 0 to 100. A lower score for a functional or global QOL scale reflects a poorer functioning level or global QOL, while a lower score for a symptom/problem scale reflects less symptoms/problem (better QOL).
European Organization for Research and Treatment of Cancer QLQ-HCC18 (EORTC QLQ-HCC18) [33]	EORTC QLQ-HCC18 includes eighteen multiple item scales organized into six domains (fatigue, body image, jaundice, nutrition, pain and fever) and two items (abdominal swelling and sex life) [33]. All scales are grouped and transformed to score ranging from 0 to 100. A lower score represents a less severe symptom/problem (better QOL). EORTC QLQ-HCC18 is used together with EORTC QLQ-C30.
C30 index score [30]	∑ [(100-Physical functioning), (100-Role functioning), (100-Emotional functioning), (100-Cognitive functioning), (100-Social functioning), (100-global QOL), scores of Fatigue, Nausea and vomiting, Pain, Dyspnoea, Insomnia, Appetite loss, Constipation, Diarrhea, Financial Diffculty] ÷ 15. (remarks: a lower score represents a less severe symptom/problem)
HCC18 index score [30]	∑(scores of Fatigue, Body Image, Jaundice, Nutrition, Pain, Fever, Sex life, Abdominal distension) ÷ 8. (remarks: a lower score represents a less severe symptom/problem)

*HCC* Hepatocellular carcinoma, *QOL* Health related quality of life

### Clinical factors

Demographic, clinical and laboratory data were also collected on the day of study entrance. Since there was no prior information on the relationship between QOL and liver function in HCC patients, we explored a broad array of liver function variables in the analyses. These included albumin, ALP, alanine transaminase (ALT), bilirubin, INR and platelet counts. Presence or absence of ascites was noted. Child-Pugh class, MELD, ALBI grade, albumin-to-ALP ratio and ALP-to-platelet ratio were derived (see Table 2).

**Table 2:** Scoring systems for liver function used in the study

ALBI score	−0.085 × albumin + 0.66 × log bilirubin (remarks: albumin in g/L, bilirubin in μmol/L)
MELD score	[9.57 x ln(Creatinine ÷ 88.4)] + [3.78 x ln(Bilirubin ÷17.1)] + [11.2 x ln(INR)] + 6.43 (remarks: bilirubin in umol/L, creatinine in umol/L)
Albumin-to-ALP ratio	albumin ÷ ALP (remark: albumin in g/L, ALP in iu/L)
ALP-to-platelet ratio	ALP ÷ platelet (remarks: ALP in iu/L, platelet count in 10^9^/L)

*ALBI* Albumin-Bilirubin, *MELD* Model for End-stage Liver Disease, *QOL* Quality of life, *ln* natural logarithm

### Statistical analysis

Standard descriptive analyses were performed to assess sample characteristics. The following liver function assessments based on stratified scoring systems were dichotomized into normal versus abnormal groups: Child-Pugh class (‘A’ versus ‘B and C’), ALBI grade (‘1’ versus ‘2 and 3’), MELD grade (‘1’ versus ‘2 and 3’), respectively. Univariate logistic regressions were used to evaluate the correlations between dichotomized liver function variables and continuous QOL factors. To control for clinical factors, baseline clinical variables including age, gender, performance status, liver biochemistry, AFP level, presence of cirrhosis, etiology of cirrhosis, tumor stage and planned treatment, together with all significant QOL variables obtained from univariate logistic model were entered into the multivariate logistic regressions with stepwise selection. For all logistic regressions, odds ratios (OR) were calculated with odds of patients belonging to abnormal liver function group against normal liver function group. Ninety-five percent confidence intervals (95% CI) of OR were also calculated. Since the QOL data were not normally distributed, the correlations between continuous liver function parameters (in natural logarithm) and the QOL data were assessed using Spearman’s rank correlation analysis. A *p*-value of less than 0.05 was accepted as statistically significant. Further, we defined Spearman’s rank correlation outcomes with coefficient (rho) of ≥0.3 or ≤ − 0.3 as potentially clinically important [34–37]. The statistical analyses were performed using statistical software (SAS version 9.3; SAS institute, Cary, NC, USA).

### Sample size estimation

Assuming Spearman’s rho between QOL and liver function variables to be at least 0.3, with two sided alpha-level of 0.05 and power of 0.9, the required sample size was 133 patients [38]. We aimed to obtain C30 and HCC18 index-scores for all analyzed patients. This required all patients to have all QOL questions answered. For this reason complete-case analysis would be preferred in the study in order to calculate index-scores accurately. To minimize the impact of complete-case analysis, we set the final target sample size to be three times the original sample size (399 patients).

## Results

### Patient characteristics

Five hundred and seventeen patients were consented, amongst whom 472 (91%) had complete QOL data and were included for analysis. Tables 3 and 4 showed the clinical characteristics and baseline QOL data of these patients. The median age at diagnosis was 60. Ninety-six percent had Eastern Cooperative Oncology Group (ECOG) performance status of 0 or 1. Sixty-eight percent of all patients were of Child-Pugh class A. Fifty-nine percent had cirrhosis. HBV infection was present in 82%, while 6% had HCV infection. One hundred and eight (23%) patients had extra-hepatic metastasis. 152 patients (32%) had portal vein thrombosis.

**Table 3:** Baseline characteristics of in the 472 HCC patients

Variable	N	%	Mean ± SD
Clinical data			
Age < = 65	328	69	60 ± 12
Male gender	419	89	
ECOG			
0	144	31	
1	299	63	
≥ 2	29	6	
Cirrhosis (radiological)	278	59	
Tumor morphology			
Uninodular	122	26	
Multinodular	143	30	
Diffuse	207	44	
Extrahepatic metastasis (nodal or distant)	108	23	
Portal vein thrombosis	152	32	
Hepatitis B surface antigen +	386	82	
Hepatitis C antibody +	30	6	
α-feto protein ≥200 mg/ml	250	53	
ALBI score	472		−2.29 ± 1.33
Grade 1	155	32.8	
Grade 2	265	56.2	
Grade 3	52	11.0	
MELD score	472		9.08 ± 4.05
Grade 1 (< 10)	319	67.6	
Grade 2 (10–14)	109	21.4	
Grade 3 (> 14)	52	11.0	
Child-Pugh class			
A	319	67.6	
B	130	27.5	
C	23	4.9	
Albumin	472	100	37.4 ± 15.2
Bilirubin	472	100	32.8 ± 45.7
INR	472	100	1.15 ± 0.17
ALP	472	100	193.4 ± 155.8
ALT	472	100	77.3 ± 69.4
Albumin-to-ALP ratio	472	100	0.29 ± 0.22
ALP-to-platelet ratio	472	100	1.20 ± 1.19
Presence of ascites	122	25.8	
Hemoglobin < 10 g/dL	27	6	
White cell count > 10 × 10^9^/L	64	14	
Platelet count < 100 × 10^9^/L	33	7	
1st line Treatment			
Surgical treatment	54	12	
Local ablative therapies	29	6	
Trans-arterial therapies	116	25	
Systemic therapies	91	19	
Best supportive care alone	182	38	

*EORTC* European Organization for Research and Treatment of Cancer, *ECOG* Eastern Cooperative Oncology Group, *ALBI* Albumin-bilirubin, *MELD* Model for End-stage Liver Disease, *INR* international normalized ratio, *ALP* alkaline phosphatases, *ALT* alanine transaminase

**Table 4:** Spearman’s rank correlation analyses between QOL and continuous liver function variables

	mean ± SD	ln(Albumin)		ln(Bilirubin)		ln(INR)		ln(ALP)		ln(ALT)		ln(Alb/ALP)		ln(ALP/plt)	
		rho	*p*	rho	*p*	rho	*p*	rho	*p*	rho	*p*	rho	*p*	rho	*p*
EORTC QLQ-C30															
Physical functioning	72.27 ± 23.74	0.404	< 0.001	− 0.235	< 0.001	−0.202	< 0.001	− 0.271	< 0.001	− 0.030	0.514	0.337	< 0.001	− 0.145	0.002
Role functioning	74.61 ± 32.60	0.318	< 0.001	− 0.262	< 0.001	− 0.194	< 0.001	− 0.304	< 0.001	− 0.187	< 0.001	0.350	< 0.001	− 0.141	0.002
Emotional functioning	70.67 ± 25.48	− 0.023	0.612	− 0.055	0.232	− 0.018	0.693	− 0.076	0.099	− 0.054	0.239	0.066	0.154	−0.044	0.345
Cognitive functioning	76.80 ± 24.68	0.133	0.004	−0.115	0.013	−0.146	0.002	−0.072	0.120	−0.029	0.533	0.094	0.041	−0.071	0.121
Social functioning	68.46 ± 30.33	0.151	0.001	−0.147	0.001	−0.116	0.012	−0.235	< 0.001	− 0.069	0.135	0.241	< 0.001	−0.082	0.076
Global quality of life	52.22 ± 26.34	0.257	< 0.001	−0.232	< 0.001	−0.167	< 0.001	− 0.230	< 0.001	−0.025	0.595	0.265	< 0.001	−0.070	0.131
Fatigue	42.93 ± 30.23	−0.321	< 0.001	0.285	< 0.001	0.246	< 0.001	0.316	< 0.001	0.127	0.006	−0.354	< 0.001	0.163	< 0.001
Nausea & vomiting	11.26 ± 21.41	−0.114	0.013	0.180	< 0.001	0.1406	0.002	0.178	< 0.001	0.093	0.043	−0.183	< 0.001	0.017	0.721
Pain	32.87 ± 31.97	−0.106	0.022	0.066	0.152	0.084	0.069	0.238	< 0.001	0.050	0.278	−0.229	< 0.001	− 0.003	0.953
Dyspnoea	29.73 ± 31.46	−0.244	< 0.0001	0.171	< 0.001	0.153	0.001	0.166	< 0.001	0.020	0.661	−0.201	< 0.001	0.111	0.016
Insomnia	41.88 ± 36.41	−0.165	< 0.001	0.110	0.017	0.104	0.024	0.215	< 0.001	0.111	0.016	−0.228	< 0.001	0.077	0.095
Appetite loss	32.34 ± 35.88	−0.200	< 0.001	0.245	< 0.001	0.134	0.004	0.300	< 0.001	0.086	0.061	−0.304	< 0.001	0.071	0.121
Constipation	16.67 ± 27.13	−0.057	0.217	0.006	0.890	−0.007	0.885	−0.019	0.684	0.024	0.598	0.008	0.857	−0.048	0.297
Diarrhea	16.45 ± 26.87	−0.164	< 0.001	0.222	< 0.001	0.146	0.001	0.126	0.006	0.119	0.009	−0.158	< 0.001	0.143	0.002
Financial difficulties	51.20 ± 37.22	−0.093	0.044	0.085	0.066	0.086	0.061	0.159	< 0.001	0.062	0.178	−0.163	< 0.001	0.077	0.093
EORTC QLQ-HCC18															
Fatigue	35.23 ± 25.86	−0.305	< 0.001	0.258	< 0.001	0.209	< 0.001	0.295	< 0.001	0.122	0.008	−0.329	< 0.001	0.148	0.001
Body Image	25.35 ± 22.98	−0.280	< 0.001	0.255	< 0.001	0.152	0.001	0.287	< 0.001	0.064	0.164	−0.320	< 0.001	0.106	0.021
Jaundice	23.41 ± 22.15	−0.094	0.041	0.226	< 0.001	0.110	0.017	0.096	0.038	0.082	0.074	−0.101	0.027	0.126	0.006
Nutrition	26.96 ± 21.35	−0.225	< 0.001	0.276	< 0.001	0.149	0.001	0.341	< 0.001	0.134	0.004	−0.343	< 0.001	0.136	0.003
Pain	23.34 ± 24.57	−0.077	0.096	0.144	0.002	0.094	0.042	0.182	< 0.001	0.057	0.217	−0.178	< 0.001	0.038	0.407
Fever	6.60 ± 14.39	−0.220	< 0.001	0.117	0.011	0.193	< 0.001	0.097	0.036	0.016	0.735	−0.131	0.004	0.041	0.378
Sex life	28.74 ± 34.76	−0.053	0.249	0.095	0.040	0.129	0.005	0.170	< 0.001	0.041	0.379	−0.157	< 0.001	0.166	< 0.001
Abdominal swelling	33.33 ± 35.43	−0.312	< 0.001	0.325	< 0.001	0.225	< 0.001	0.299	< 0.001	0.108	0.019	−0.343	< 0.001	0.175	0.001
Index-scores															
C30 index-score	30.69 ± 19.61	−0.272	< 0.001	0.250	< 0.0001	0.1927	< 0.001	0.301	< 0.0001	0.1245	0.007	−0.329	< 0.001	0.116	0.011
HCC18 index-score	25.37 ± 17.21	−0.304	< 0.001	0.318	< 0.0001	0.2229	< 0.001	0.343	< 0.0001	0.1188	0.010	−0.371	< 0.001	0.187	< 0.001

*ALP* alkaline phosphatase, *ALT* alanine transaminase, *EORTC* European Organization for Research and Treatment of Cancer, *INR* international normalized ratio, *ln* natural logarithm, *MELD* Model for End-stage Liver Disease, *Plt* platelet, *rho* Spearman’s rank correlation coefficient

### Correlations between QOL and liver biochemistries

**Fig. 1:**
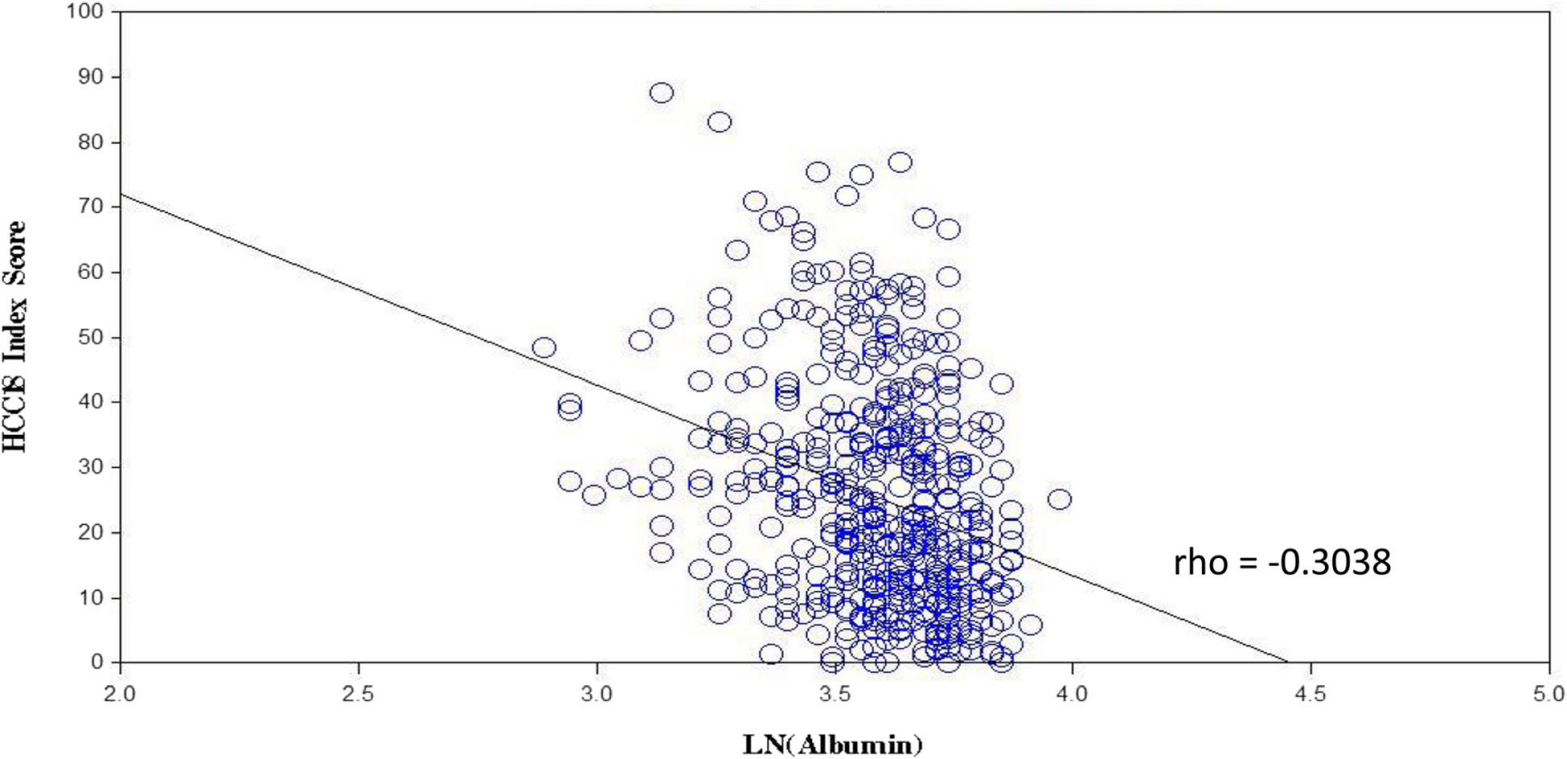
Scatter plot of HCC1 8 index-score against ln(albumin level). ln – natural logarithm; rho – Spearman’s rank correlation coefficient

Albumin level had significant correlations with C30 index-score, HCC18 index-score, and majority of scales in QLQ-C30 and QLQ-HCC18 (see Table 4). A higher albumin level was correlated with a better QOL. Among these correlations, six were considered to be potentially clinically important, namely QLQ-C30 ‘physical functioning’, ‘role functioning’, ‘fatigue’, QLQ-HCC18 ‘fatigue’, ‘abdominal swelling’ and HCC18 index-score. Figure 1 shows the scatter plot of HCC18 index-score against albumin level.

**Fig. 2:**
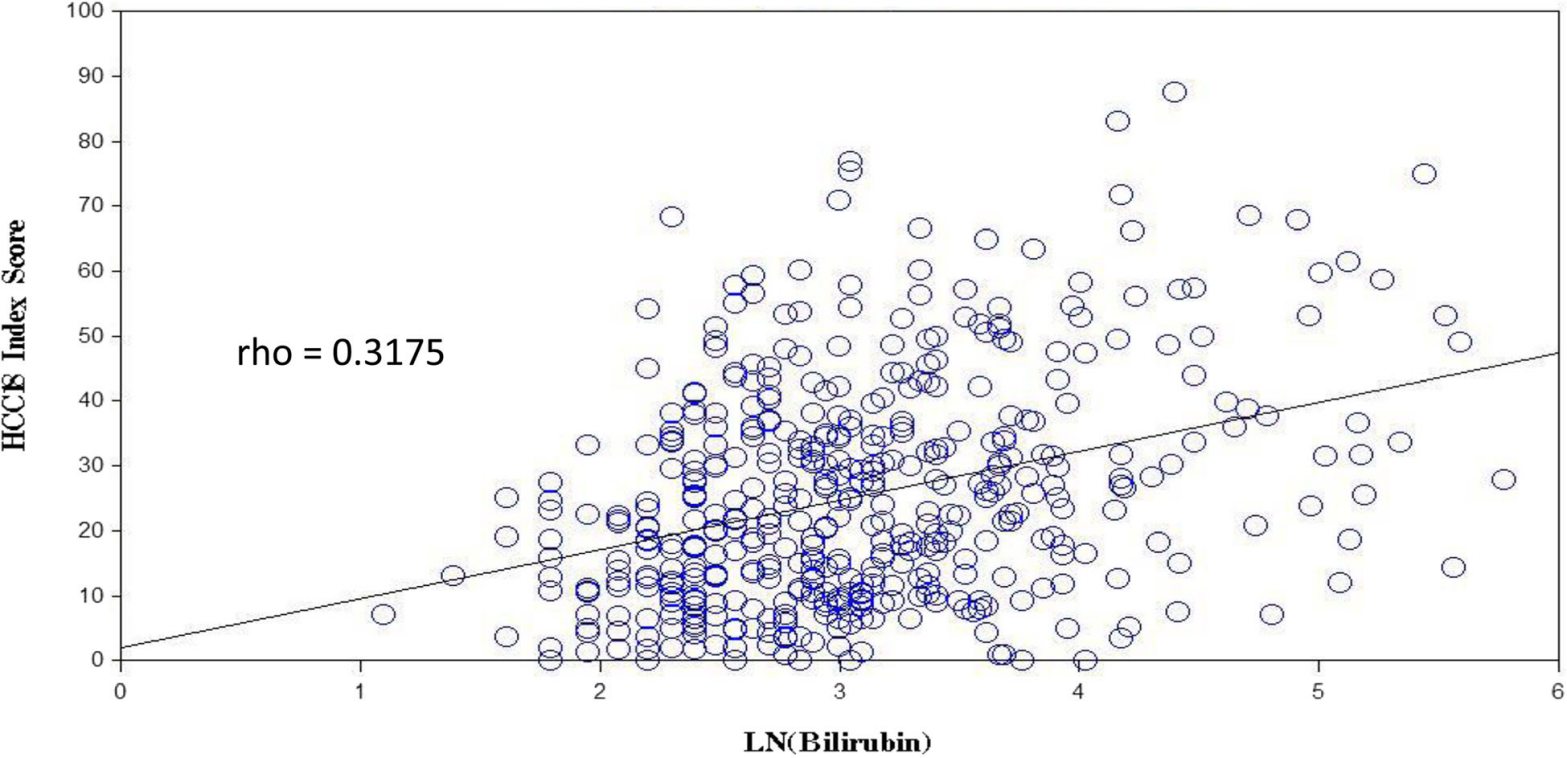
Scatter plot of HCC 18 index-score against ln(bilirubin level). ln – natural logarithm; rho – Spearman’s rank correlation coefficient

Bilirubin level had significant correlations with C30 index-score, HCC18 index-score, majority of scales in QLQ-C30 and QLQ-HCC18 (see Table 4). The lower the bilirubin level, the better the QOL. Two correlations, QLQ-HCC18 ‘abdominal swelling’ and HCC18 index-score, were potentially clinically important. Figure 2 shows the scatter plot of HCC18 index-score versus bilirubin level.

INR level had significant correlations with C30 index-score, HCC18 index-score, most scales in QLQ-C30 and all the eight scales in QLQ-HCC18 (Table 4). Lower INR level was correlated with better QOL. A significant and relatively stronger correlation was seen with QLQ-C30 ‘fatigue’; however, the Spearman’s rho was only 0.25 and thus, there was no correlation identified to be potentially clinically important.

**Fig. 3:**
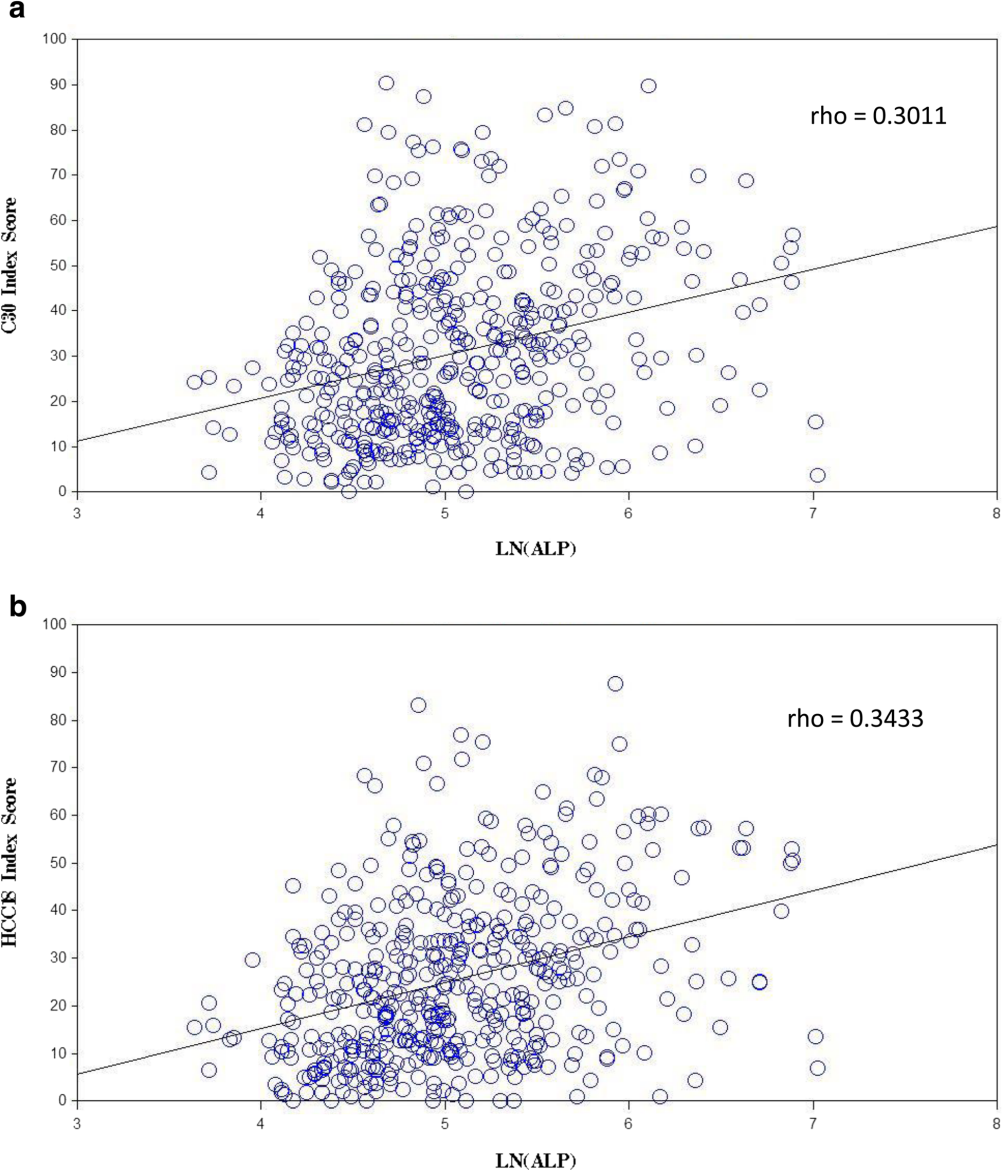
**a**. Scatter plot of C30 index-score against ln(alkaline phosphatase level). **b** Scatter plot of HCC1 8 index-score against ln(alkaline phosphatase level). ln – natural logarithm; rho – Spearman’s rank correlation coefficient

ALP level had significant correlations with C30 index-score, HCC18 index-score, most scales in QLQ-C30 and all QLQ-HCC18 scales (Table 4). Patients with lower ALP had better QOL. Five correlations were potentially clinically important: HCC18 index-score, C30 index-score, QLQ-HCC18 nutrition, QLQ-C30 ‘fatigue’ and ‘role functioning’. Figure 3a and b show the scatter plots of C30 and HCC18 index-scores against ALP level respectively.

ALT level had significant correlations with C30 index-score, HCC18 index-score, a few scales in QLQ-C30 and QLQ-HCC18 (Table 4). However, none of these correlations was considered to be potentially clinically important.

### Correlation between QOL and albumin-to-ALP ratio

**Fig. 4:**
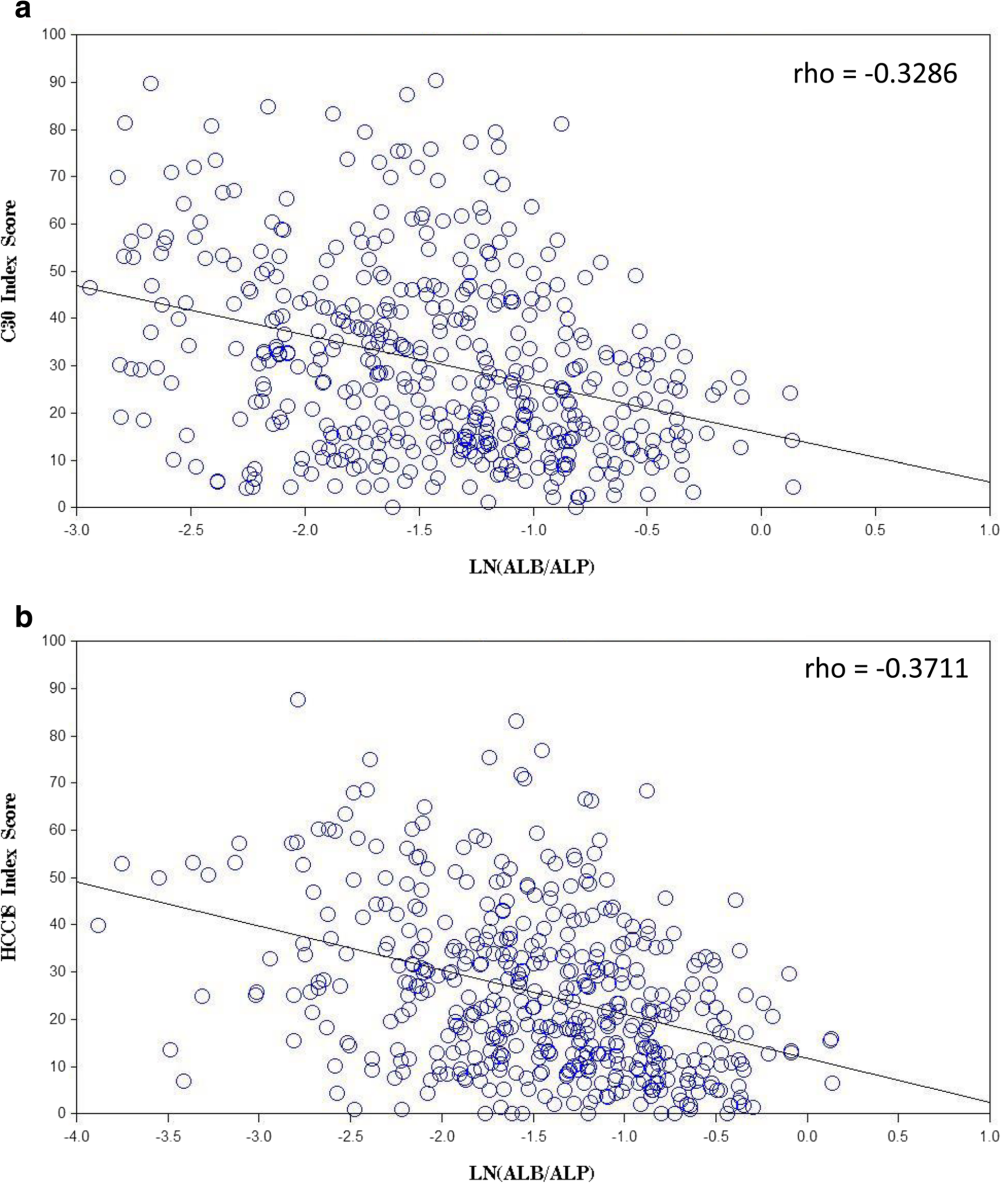
**a**. Scatter plot of C30 index-score against ln(albumin to alkaline phosphatase ratio). **b** Scatter plot of HCC1 8 index-score against ln(albumin to alkaline phosphatase ratio). ln – natural logarithm; rho – Spearman’s rank correlation coefficient

Albumin-to-ALP ratio had significant correlations with C30 index-score, HCC18 index-score, majority of scales in QLQ-C30 and all eight scales in QLQ-HCC18 (see Table 4). A better albumin-to-ALP ratio was associated with a better QOL. Ten potentially clinically important correlations were identified: HCC18 index-score, C30 index-score, QLQ-HCC18 ‘nutrition’, ‘abdominal swelling’, ‘fatigue’, ‘body image’, QLQ-C30 ‘fatigue’, ‘role functioning’, ‘physical functioning’ and ‘appetite loss’. Figure 4a and b show the scatter plots of C30 and HCC18 index-scores against albumin-to-ALP ratio respectively.

### Correlation between QOL and ALP-to-platelet ratio

ALP-to-platelet ratio had significant correlations with C30 index-score, HCC18 index-score, a few scales in QLQ-C30 and QLC-HCC18 (see Table 4). However, none of these correlations was high enough to be regarded as potentially clinically important.

### Correlation between QOL and child-Pugh class

Thirty-two percent of patients were in Child’s class B or C, the rest were in Child’s class A (see Table 3). There were significant correlations between Child’s class and QOL variables in univariate logistic regressions (see Tables 5). Patients with worse C30 index-score, HCC18 index-score, as well as worse QOL scores in majority of scales in QLQ-C30 and QLQ-HCC18 were more likely to be in Child’s class B or C (*p* < 0.01). After adjusting for clinical variables, QOL remained significantly correlated with Child’s class (see Table 6).

**Table 5:** Univariate logistic regressions of health related quality of life variables for abnormal categorical liver function variables

Variable Name	Child’s classes B to C			ALBI grades 2 to 3			Presence of ascites			MELD grades 2 to 3		
	OR	95% CI	*p*-value	OR	95% CI	*p*-value	OR	95% CI	*p*-value	OR	95% CI	*p*-value
EORTC QLQ-C30												
Physical functioning	0.972	0.964–0.981	< 0.001	0.962	0.952–0.973	< 0.001	0.974	0.965–0.982	< 0.001	0.989	0.981–0.997	0.009
Role functioning	0.982	0.976–0.987	< 0.001	0.975	0.967–0.983	< 0.001	0.982	0.976–0.988	< 0.001	0.992	0.986–0.998	0.007
Emotional functioning	0.997	0.990–1.005	0.508	0.997	0.989–1.004	0.396	0.995	0.988–1.003	0.262	1.002	0.994–1.010	0.597
Cognitive functioning	0.988	0.980–0.996	0.002	0.987	0.979–0.996	0.003	0.991	0.980–1.001	0.082	0.993	0.985–1.000	0.064
Social functioning	0.990	0.984–0.996	0.002	0.986	0.979–0.993	< 0.001	0.989	0.982–0.996	0.001	0.995	0.989–1.001	0.124
Global quality of life	0.980	0.972–0.987	< 0.001	0.978	0.970–0.986	< 0.001	0.979	0.970–0.987	< 0.001	0.989	0.981–0.996	0.003
Fatigue	1.022	1.015–1.029	< 0.001	1.025	1.017–1.033	< 0.001	1.025	1.017–1.032	< 0.001	1.010	1.003–1.016	0.003
Nausea & vomiting	1.014	1.005–1.023	0.002	1.019	1.007–1.031	0.001	1.013	1.004–1.022	0.004	1.009	1.001–1.018	0.037
Pain	1.005	0.999–1.011	0.099	1.008	1.002–1.015	0.010	1.007	1.000–1.013	0.036	0.998	0.992–1.004	0.479
Dyspnoea	1.016	1.009–1.022	< 0.001	1.016	1.009–1.023	< 0.001	1.017	1.010–1.024	< 0.001	1.007	1.001–1.013	0.033
Insomnia	1.009	1.004–1.015	0.001	1.010	1.005–1.016	< 0.001	1.010	1.005–1.016	0.001	0.999	0.993–1.004	0.638
Appetite loss	1.014	1.008–1.019	< 0.001	1.016	1.010–1.023	< 0.001	1.015	1.009–1.020	< 0.001	1.007	1.002–1.012	0.010
Constipation	1.004	0.998–1.011	0.205	1.002	0.995–1.010	0.508	1.007	0.999–1.014	0.072	0.998	0.991–1.005	0.586
Diarrhea	1.017	1.009–1.024	< 0.001	1.016	1.007–1.025	0.003	1.017	1.010–1.025	< 0.001	1.012	1.005–1.019	0.001
Financial difficulties	1.003	0.998–1.008	0.253	1.007	1.002–1.012	0.011	1.004	0.998–1.009	0.200	1.000	0.995–1.006	0.861
EORTC QLQ-HCC18												
Fatigue	1.024	1.016–1.032	< 0.001	1.030	1.020–1.040	< 0.001	1.022	1.013–1.030	< 0.001	1.008	1.001–1.016	0.027
Body Image	1.029	1.020–1.038	< 0.001	1.025	1.015–1.035	< 0.001	1.034	1.024–1.044	< 0.001	1.014	1.005–1.022	0.001
Jaundice	1.024	1.015–1.033	< 0.001	1.011	1.002–1.021	0.020	1.016	1.007–1.025	0.001	1.018	1.009–1.027	< 0.0001
Nutrition	1.027	1.017–1.037	< 0.001	1.026	1.015–1.037	< 0.001	1.024	1.014–1.033	< 0.001	1.013	1.004–1.022	0.003
Pain	1.012	1.004–1.020	0.003	1.013	1.004–1.022	0.004	1.013	1.005–1.022	0.001	1.001	0.993–1.009	0.802
Fever	1.023	1.009–1.037	0.001	1.033	1.014–1.053	0.001	1.012	0.999–1.026	0.079	1.011	0.998–1.025	0.085
Sex life	1.004	0.999–1.010	0.130	1.007	1.001–1.013	0.017	1.002	0.997–1.008	0.431	1.005	0.999–1.010	0.089
Abdominal swelling	1.023	1.017–1.029	< 0.001	1.019	1.012–1.026	< 0.001	1.028	1.022–1.035	< 0.001	1.009	1.004–1.015	0.001
Index-scores												
C30 index-score	1.029	1.019–1.040	< 0.001	1.037	1.024–1.049	< 0.001	1.031	1.020–1.042	< 0.001	1.011	1.001–1.021	0.028
HCC18 index-score	1.045	1.032–1.058	< 0.001	1.044	1.029–1.058	< 0.001	1.043	1.030–1.056	< 0.001	1.020	1.009–1.032	< 0.001

*ALBI* albumin-bilirubin, *CI* Confidence intervals, *EORTC* European Organization for Research and Treatment of Cancer, *MELD* Model for End-stage Liver Disease, *OR* Odds ratio

**Table 6:** Multivariate logistic regressions of clinical and health related quality of life variables for abnormal categorical liver function variables

	Child’s classes B to C			ALBI grades 2 to 3			Presence of ascites			MELD grades 2 to 3		
	OR	95% CI	*p*-value	OR	95% CI	*p*-value	OR	95% CI	*p*-value	OR	95% CI	*p*-value
EORTC QLQ-C30 Physical Functioning	0.987	0.974–1.000	0.045	0.973	0.961–0.985	< 0.001	–	–	–	–	–	–
EORTC QLQ-HCC18 Abdominal Swelling	1.012	1.003–1.021	0.011	–	–	–	1.020	1.012–1.028	< 0.001	–	–	–
CUPI	4.239	2.357–7.626	< 0.001	2.580	1.557–4.277	< 0.001	10.538	5.944–18.681	< 0.001	–	–	–
Bilirubin	1.100	1.073–1.126	< 0.001	1.089	1.058–1.121	< 0.001	1.010	1.001–1.019	0.029	1.093	1.072–1.114	< 0.001
ln(AFP)	0.838	0.763–0.920	0.001	–	–	–	0.790	0.721–0.865	< 0.001	–	–	–
Planned treatment modality	–	–	–	2.149	1.162–3.972	0.015	–	–	–	–	–	–

*ALBI* albumin-bilirubin, *CI* Confidence intervals, *CUPI* the Chinese University Prognostic Index, *EORTC* European Organization for Research and Treatment of Cancer; *ln(AFP)* natural logarithm of alpha-feto protein level, *MELD* Model for End-stage Liver Disease, *OR* Odds ratio

### Correlation between QOL and ALBI grade

Three hundred and seventeen patients (67%) were classified as ALBI grades 2–3, 155 (33%) as ALBI grade 1 (Table 3). QOL had significant correlations with ALBI grade (see Table 5). Patients with worse C30 index-score, HCC18 index-score, as well as worse scores in majority of scales in QLQ-C30 and all the eight scales in QLQ-HCC18 were more likely to be of ALBI grade 2 or 3 (*p* < 0.03). After adjusting for clinical variables, QOL remained significantly correlated with ALBI grade (see Table 6).

### Correlation between QOL and the presence of ascites

One hundred and twenty two patients (26%) presented with ascites at diagnosis (Table 3). QOL was significantly correlated with the presence of ascites (see Table 5). Patients with worse C30 index-score, HCC18 index-score, as well as worse QOL scores in majority of scales in QLQ-C30 and QLQ-HCC18 were more likely to have ascites (*p* < 0.05). After adjusting for clinical variables, QOL remained significantly correlated with ALBI grade (see Table 6).

### Correlation between QOL and MELD

One hundred and sixty one patients (32.4%) were of MELD grade 2 or 3, while 319 (67.6%) were of grade 1 (see Table 3). There were significant correlations between MELD grade and QOL variables in univariate logistic regressions (see Tables 5). Patients with worse C30 index-score, HCC18 index-score, as well as worse scores in majority of scales in QLQ-C30 and QLQ-HCC18 were more likely to be of MELD grade 2 or 3 (p < 0.05). However, after controlling for clinical variables, no QOL variable was significantly correlated with MELD grade (see Table 6).

## Discussion

This is the first report on the correlation between QOL and baseline liver function in patients with HCC. QOL assessments with EORTC QLQ-C30 and QLQ-HCC18 had significant correlations with most of the continuous liver function parameters evaluated. The correlations with levels of albumin, bilirubin, ALP and albumin-to-ALP ratio were potentially clinically important (where Spearman’s rho were ≥ 0.3 or ≤ − 0.3). After adjusting for clinical variables, QOL was also demonstrated to have significant correlations with dichotomized liver function factors, including Child’s class, ALBI grade and the presence of ascites,. The strongest single correlation in this study was between QLQ-C30 ‘physical functioning’ and albumin level, where the Spearman’s rho was 0.40. On the other hand, albumin-to-ALP ratio had the highest number (a total of ten) of potentially clinically important correlations with QOL.

In our earlier study, we have developed C30 and HCC18 index-scores in an attempt to simplify the various domains and items scores in the EORTC QLQ-C30 and QLQ-HCC18 tools respectively for survival analyses, and they were found to be significant prognostic factors for OS [30]. We proposed to use these 2 respective index-scores as they are easy to calculate and could be conducted in daily clinic setting. In the current analysis, we aimed to assess the correlations between QOL and liver function in HCC patients. Without prior knowledge on the level of correlation with various QOL factors, we have included the available index-scores alongside the ‘standard’ QOL domains and items in the analyses in order to assess how well each of these could perform. Findings from the current analyses support the fact that the 2 index-scores have potentially clinically important correlations with four continuous liver function variables. None-the-less, it is acknowledged that these indices are by no means able to replace the domains and items within QLQ-C30 and QLQ-HCC18 that addressed QOL in greater depth.

The current study has some limitations. Since the sample size of this study was large enough, even what appeared to be relatively weak correlations have been shown to have statistically significant *p*-values. For examples, a number of QOL factors showed significant correlations with ALT level and ALP-to-platelet ratio. However, it has to be noted that the magnitudes of these Spearman’s rho were too weak and thus they are unlikely to have clinical importance. Another limitation would be the lack of follow up assessments of liver function and QOL. These data could be potentially useful, since correlation analyses between QOL and liver function at later time-points may further depict the relationships between liver function and QOL. Thus, further studies with longitudinal follow-up data are warranted.

QOL has gained increasing attention in HCC patient management; specifically, improving patients’ QOL has become an important goal to clinicians. Most phase III clinical trials in HCC patients reported QOL as one of the main study endpoints [39–43]. QOL in HCC patients is known to be related to the tumor severity and treatment toxicity. The current study reported potentially clinically important correlations between QOL and liver function in HCC patients. The study findings highlight to clinicians the relevance of liver function in addition to tumor burden to QOL among HCC patients. Along with HCC tumor per se, liver functional impairment may be the result of other co-existing conditions including viral infections and biliary obstruction. Interventional treatments, by means of anti-viral drug administration and radiological or surgical biliary drainage respectively might improve liver function and have positive impact on QOL of HCC patients. Future trials are warranted to assess whether treatment to enhance liver function could improve HCC patients’ QOL.

## Conclusions

With the background knowledge that QOL and liver function are both independent prognostic factors for survival in HCC patients irrespective of stage, this study further explored the relationship between QOL and liver function. In HCC patients, baseline QOL assessment (using EORTC QLQ-C30, QLQ-HCC18, C30 index-score or HCC18 index-score) is significantly correlated with liver function. Based on the findings of this study, future trials are warranted to assess whether treatment to enhance liver function could improve HCC patients’ QOL.

## Abbreviations

AFP: Alpha fetoprotein
ALBI: Albumin-bilirubin
ALP: Alkaline phosphatase
ALT: Alanine transaminase
CI: 95% confidence intervals
CLDQ: Chronic Liver Disease Questionnaire
ECOG: Eastern Cooperative Oncology Group
EORTC: European Organization for Research and Treatment of Cancer
FACT-G: Functional Assessment of Cancer Therapy - General
HBV: Hepatitis B virus
HCC: Hepatocellular carcinoma
HCV: Hepatitis C virus
INR: International normalized ratio
MELD: Model for End-stage Liver Disease
OR: Odds ratio
OS: Overall survival
QOL: Quality of life
rho: Spearman’s rank correlation coefficient
